# Influence of bacterial morphotype on urine culture and molecular epidemiological differences in *Escherichia coli* harboring bacterial morphotype-induced urinary tract infections

**DOI:** 10.1128/spectrum.00980-24

**Published:** 2025-03-05

**Authors:** Yewei Fang, Shuan Tao, Huimin Chen, Yao Xu, Luyan Chen, Wei Liang

**Affiliations:** 1 Department of Clinical Laboratory, The First Affiliated Hospital of Ningbo University 117881 , Ningbo, China; 2 School of Medicine, Jiangsu University, Zhejiang, China; 3 School of Medicine, Ningbo University 47862 , Ningbo, China; 4 Department of Blood Transfusion, The First Affiliated Hospital of Ningbo University 117881 , Ningbo, China; MultiCare Health System, Tacoma, Washington, USA

**Keywords:** *E. coli*, urinary tract infection, bacterial morphotype, virulence, urine culture, whole genome sequencing

## Abstract

Bacteria that adhere to epithelial cells, form intracellular bacterial communities (IBCs), or transition to filamentous forms are referred to as morphotype-positive bacteria. *Escherichia coli* (*E. coli*) with this morphotype plays a critical role in urinary tract infections (UTIs), yet its impact on urine culture outcomes and molecular epidemiological characteristics remains unclear. In this retrospective study, we assessed the effect of bacterial morphotype on urine culture results and investigated the molecular differences between *E. coli* strains with and without this morphotype, using PCR and whole genome sequencing (WGS). We observed that *E. coli* with the morphotype-positive phenotype frequently appeared in urine sediments, leading to fewer colony-forming units (CFUs) in culture and contributing to false-negative results. However, vortexing the urine samples significantly increased CFUs, improving culture sensitivity. Additionally, *E. coli* with the positive morphotype carried more adhesion-related virulence genes (VGs), with the majority belonging to phylogenetic group B2. Whole genome sequencing further revealed a broader array of virulence genes in these strains. Our findings demonstrate that vortexing is an effective method to enhance urine culture positivity by releasing intracellular bacteria, and that morphotype-positive *E. coli* harbors a diverse set of virulence factors, indicating their potential high pathogenicity. These results highlight the importance of detecting bacterial morphotypes in urine samples for accurate UTI diagnosis and emphasize the need for increased attention to these highly virulent strains.

**IMPORTANCE:**

Uropathogenic *Escherichia coli* (UPEC) is widely acknowledged as the primary pathogen responsible for urinary tract infections (UTIs). Following adherence to the epithelium, UPEC undergoes periodic morphological changes, such as filamentation, which not only contribute to immune evasion but also lead to false-negative results. This study focuses on three transient stages of UPEC morphological changes: adherence to the epithelium, formation of intracellular bacterial communities (IBCs), and the presence of filamentous UPEC. Any one of these characteristics is acceptable to classify UPEC strains as morphotype-positive UPEC. This study reported the prevalence of UPEC with the bacterial morphotype and established a direct relationship between urine culture and bacterial morphotype. The molecular epidemiological distinctions were both revealed. These findings provide further evidence of the necessary for bacterial morphotype detection, and greater attention should be given to *E. coli* harboring this bacterial morphotype.

## INTRODUCTION

**Fig 1:**
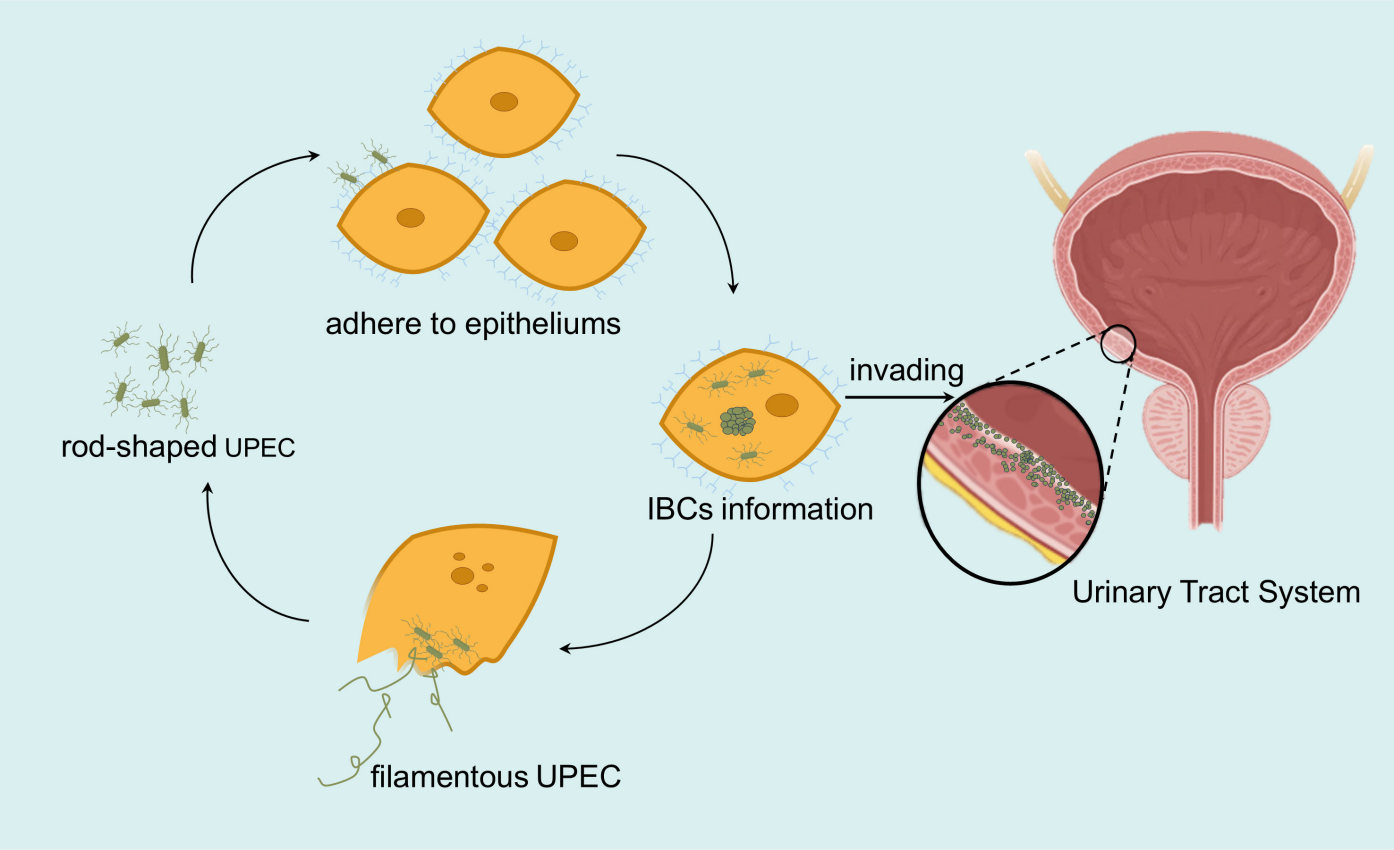
A mode of morphological transformation cycle of MP UPEC. This mode includes the following three stages of MP UPEC invasion of the urine epithelium: UPEC initially adopts a rod-shaped form, adheres to epithelial cells, and penetrates them through fimbriae and other adhesins. It then replicates extensively within these cells, transitioning from a dispersed rod-shaped to a dense, clustered, spherical form known as IBCs. Once the host epithelial cells perish, numerous UPEC are released into the external environment in a filamentous form. These UPEC then revert to their original rod-shaped form, seeking new epithelia of the urinary tract to adhere to, thus completing a full cycle of morphological transformation, leading to a constant invasion of the urinary tract system. UPEC, uropathogenic *Escherichia coli*; IBC, intracellular bacterial community; MP, morphotype positive.

Statistical analyses indicate that globally, approximately 250 million individuals are affected by urinary tract infections (UTIs) (1), leading to an estimated economic loss of around 3.5 billion dollars in the USA alone (2). Roughly 40% of women are likely to experience at least one UTI in their lifetime (3). The recurrence of UTIs requires multiple medical consultations and the use of various antibiotics, causing significant psychological and economic strain on patients. At the same time, it also strengthened the generation and spread of clinical drug-resistant strains (4). Uropathogenic *Escherichia coli* (UPEC) is recognized as the main pathogen responsible for UTIs, accounting for about 80%–90% of cases (5). The colonization of the urinary tract epithelium by UPEC, facilitated by fimbriae and other adhesins, is a crucial step in the pathogenesis of UTIs (4). Notably, UPEC undergoes periodic morphological changes after adhering to the urinary tract epithelium (6, 7). These changes involve UPEC initially adopting a rod-shaped form, adhering to, and penetrating epithelial cells through fimbriae and other adhesins, then replicating extensively within these cells, transitioning from a dispersed rod-shaped to a dense, clustered, spherical form known as intracellular bacterial communities (IBCs). After the host epithelial cells perish, numerous UPEC are released into the external environment in a filamentous form, which then revert to their original rod-shaped form, looking for new hosts to adhere to, thus completing a full cycle of morphological transformation (8). UPEC strains exhibiting any one of the three transient stages—UPEC adherence to the epithelium, formation of IBCs, and release in filamentous UPEC—are characterized as positive bacterial morphotype phenotypes in this study (Fig. 1).

Recent studies have shown that FimH interacts with mannosylated glycoproteins on the outer layer of the urothelium, facilitating attachment and initiating bacterial entry into the bladder epithelial cells (9). These processes are followed by morphological transformations (10) which are mediated by the cell division gene *DamX, as* observed in mouse models of cystitis (8). In addition to this, IBCs and filamentous forms of UPEC were also found in the urinary sediments of patients with acute and recurrent UTIs, indicating the prevalence of positive bacterial morphotype UPEC strains (11–13). Furthermore, a significant increase in colony-forming unit (CFU) counts following the release of intracellular bacteria into the extracellular environment through vortexing during IBC observation was observed by a recent study (14), likely due to the release of intracellular bacteria. Consequently, we hypothesize that periodic morphological changes in bacteria may aid in immune evasion, making them difficult to detect in urine cultures and potentially leading to false-negative results. Moreover, bacterial adhesion plays a key role in the virulence of UPEC (15), so it is possible that bacteria with strong adhesion possess a greater number of virulence genes (VGs), particularly those related to adhesion. This can pose challenges to diagnosing and treating UTIs. Therefore, it is crucial to investigate the molecular epidemiological characteristics of positive bacterial morphotype UPEC strains and to address the low CFU counts associated with them, as this results in a lower positivity rate in urine cultures. However, previous research has not categorized the findings based on this important bacterial morphotype element, thus failing to adequately explore these concerns.

In this study, 593 urine samples were collected from patients diagnosed with UTIs at The First Affiliated Hospital of Ningbo University. Brightfield microscopy was employed to determine the bacterial morphotype, which was then used as a key criterion for grouping the urine samples into morphotype positive (MP) and morphotype negative (MN). Subsequently, the relationship between bacterial morphotype and CFU counts from urine cultures was investigated. Additionally, the urine samples were vortexed prior to bacterial culture analysis to compare CFU counts before and after vortexing. PCR techniques were performed for the detection of virulence genes, phylogenetic groups, and multilocus sequence typing (MLST). Whole genome sequencing (WGS) was allowed for the precise identification of all virulence and resistance genes, facilitating an examination of molecular epidemiological differences between the groups.

The study revealed that the urine samples from patients carrying bacterial morphotype UPEC strains showed lower CFU counts compared to the morphotype-negative group. Additionally, the positivity rate of urine cultures was increased by vortexing, leading to higher CFU values. It was observed that the morphotype-positive UPEC strains harbored a significantly greater number of virulence genes, suggesting a potential for increased pathogenicity and warranting further investigation.

## MATERIALS AND METHODS

### Study population and sample selection

This study enrolled female outpatients aged 18–65 years who presented with their first clinically confirmed episode of UTIs. To ensure that no patients with a prior history of UTIs were included, individuals with recurrent or previously documented UTIs were explicitly excluded. The decision to focus exclusively on female patients was based on both physiological and epidemiological considerations: women have a significantly higher risk of UTIs due to anatomical factors, such as a shorter urethra, which facilitates the ascension of bacteria into the bladder (16). Epidemiological data indicate that 50%–60% of women will experience at least one UTI in their lifetime, while the incidence in men is considerably lower (1, 15). By focusing on the most affected demographic, the study aims to improve the relevance and applicability of its findings.

Patients were excluded if they were male, younger than 18 years, had chronic conditions (e.g., vaginitis, diabetes, or malignant tumors) that could confound the results, or had a prior history of UTIs (including recurrent infections). Patients on long-term medication or those who refused to provide informed consent were also excluded. These criteria ensured the homogeneity of the study population and minimized potential confounding factors.

A total of 535 spontaneously voided, clean-catch midstream urine samples (one sample per patient) were randomly selected from eligible outpatients at The First Affiliated Hospital of Ningbo University, collected between November 2023 and April 2024.

### Sample handling and bacterial identification

Urine samples were processed immediately upon collection. Pathogenic strains isolated from 535 collected urine samples were identified using matrix-assisted laser desorption/ionization time-of-flight mass spectrometry (MALDI-TOF MS) (bioMérieux, Marcy l'Étoile, France) (17). For each sample, a single colony was smeared directly onto the target plate, followed by the addition of 1 µL of the matrix solution (α-cyano-4-hydroxycinnamic acid, CHCA) to the target plate. The plate was then allowed to dry at room temperature before mass spectrometry analysis. Quality control was conducted using *Escherichia coli* ATCC 25922, and all steps were performed in accordance with the manufacturer’s instructions (18).

### Urine culture and morphotype detection in urine samples

Ten milliliters of urine samples was divided into two equal portions. Ten microliters of urine from one portion (5 mL) was used for conventional urine culture, and this portion was also subjected to be vortexed immediately for 10 minutes to release the bacteria from within epithelial cells, followed by another round of urine culture. To create the control groups, *E. coli* confirmed by mass spectrometry was dissolved in sterile saline with a concentration of 0.5 McFarland standard and then diluted 1:1,024 to facilitate the counting. Although the generation time of *E. coli* is 20 to 30 minutes, the control groups were set up to exclude the effect of this 10 minute period on bacterial proliferation. The urine cultures were incubated at 37°C under aerobic conditions for 18 hours. The other portion of the urine was dedicated to detecting bacterial morphotypes. The 5 mL urine sample in the centrifuge tube was centrifuged at a force of 160 × *g* for 5 minutes to collect 0.2 mL of urinary sediment. Subsequently, 50 µL of Sternheimer-Malbin stain was added to the urinary sediment and allowed to stain for 1 minute. In this study, the morphotype-positive UPEC strains were identified through light microscopy at 40× magnification based on three distinct morphological phenomena: (i) scattered adherence*—E. coli* loosely adhering to the surface of epithelial cells in a dispersed pattern; (ii) IBCs—bacterial clusters forming inside epithelial cells, where bacteria were observed gathering in a compact formation within the cell cytoplasm; (iii) filamentous forms—elongated, filamentous *E. coli*, with an extended length compared to typical rod-shaped bacteria. UPEC strains exhibiting any of these characteristics were classified as morphotype positive, while strains lacking all three characteristics were classified as morphotype negative. When observing bacterial morphotypes under the microscope, the fine adjustment wheel was rotated to ensure that the epithelial cells and bacteria were positioned in the same focal plane. This step was critical to distinguish UPEC that was firmly adhered to or internalized within epithelial cells (morphotype positive) from free-floating bacteria that had no interaction with the epithelial cells. This adjustment was necessary to avoid misclassifying free-floating UPEC as morphotype positive and to ensure accurate identification of bacterial adherence or intracellular localization. A total of 52 UPEC strains from the MP group and 90 UPEC strains from the MN group were included in the study. Among these, 42 cases from the MP group and 58 cases from the MN group were randomly selected for further molecular epidemiological analysis.

### Detection of phylogenetic group

DNA from UPEC colonies was extracted through the boiling lysis technique (19), which included heating at 90°C for a duration of 10 minutes. Polymerase chain reaction (PCR) was executed to identify the genes *gadA*, *chuA*, *yjaA*, and *TSPE4.C2* (20). The specific primers utilized are listed in Table S1.

The PCR reaction mixture contained 0.5 µL of each 10 µmol/L forward and reverse primers, 5 µL of reaction mix reagent, 5 µL of distilled water, and 1 µL of 50–70 ng/µL DNA extract, totaling 12 µL. PCR amplification was carried out using a GeneAmp PCR System 9700 from Applied Biosystems Inc., Foster City, CA, USA. The protocol included an initial denaturation at 95°C for 5 minutes, followed by 30 cycles of 1 minute at 95°C, 1 minute at 60°C, and 5 minutes at 72°C, with a final extension at 72°C for 10 minutes. The expected sizes of the PCR products for the *gadA, chuA, yjaA*, and *TSPE4.C2* genes were 373, 281, 216, and 158 bp, respectively. The PCR products were then subjected to electrophoresis using a 1% agarose gel at 120 V for 30 minutes and were visualized under ultraviolet illumination.

The PCR results categorized the phylogenetic groups as follows: *chuA–TSPE4.C2*+ indicated group B1, *chuA+yjaA*+ represented group B2, *chuA–TSPE4.C2*– corresponded to group A, and *chuA+yjaA*– was indicative of group D. The *gadA* gene was confirmed as an inside control gene.

### Multilocus sequence typing

PCR assays were conducted on UPEC strains using a standardized protocol for MLST of *E. coli*. The amplification products were sequenced with the BigDye Terminator v3.1 Cycle Sequencing Kit, using a forward primer. The sequencing data obtained were uploaded to the PubMLST database (https://pubmlst.org/escherichia), which is an open-access platform widely used for the comparison and classification of allelic profiles and sequence types in *E. coli*.

The sequencing data were analyzed using the MLST software available on the PubMLST platform, which automatically assigns allelic profiles and sequence types (STs) based on the combination of seven housekeeping genes (*adk, fumC, gyrB, icd, mdh, purA, recA*).

The PCR amplification conditions and reagents used were consistent with those specified for previous phylogenetic group analysis, and the primer sequences for MLST (21) can be found in Table S1.

### Detection of virulence genes

PCR assays were conducted on 12 virulence genes to assess the adhesion capabilities of UPEC strains. The virulence gene profile of each isolate was determined by counting the detected genes. The selection of these virulence genes was based on previous literature (22–26), which established their relevance to bacterial adhesion of UPEC, and the primer sequences of the virulence genes are listed in Table S2.

### Susceptibility testing

The susceptibility of UPEC was assessed using a VITEK 2 Compact automated susceptibility testing system (bioMérieux, SA, France) with VITEK 2 AST-N334 cards containing a range of antibiotics, including levofloxacin (LVX), ceftriaxone (CRO), cefepime (FEP), cefuroxime (CXM), cefoxitin (FOX), ceftazidime (CAZ), imipenem (IMP), ertapenem (ETP), amoxicillin/clavulanate (AMC), cefoperazone/sulbactam (SFP), piperacillin/tazobactam (TZP), tigecycline (TGC), amikacin (AMK), and sulfamethoxazole (SXT). The drug resistance profile of the bacteria was determined according to the guidelines outlined in the M100-S32 publication by the American Clinical and Laboratory Standards Institute (CLSI) in 2022 (27). In this study, *E. coli* isolated from the urine of patients with UTI and the feces of healthy volunteers was first compared for antibiotic resistance, followed by a comparison of UPEC in the MP and MN groups.

### Whole genome sequencing

Three UPEC strains from the MP group and three UPEC strains from the MN group were randomly selected. The whole genome DNA of these strains was extracted with the TIANamp Bacteria DNA Kit (TIANGEN BIOTECH CO., LTD., Beijing, China). DNA purity and concentration were evaluated using a NanoDrop 2000 spectrophotometer (Thermo Fisher Scientific, Waltham, MA, USA). Subsequently, the DNA samples underwent library preparation and sequencing on the Illumina NovaSeq 6000 PE150 System at Hangzhou Weishu BIOTECH CO., LTD. Genome annotations were conducted using the Prokka software.

To identify antimicrobial resistance (AMR) genes in the six strains, the Abricate tool (28) was employed to compare the results against the CARD and ResFinder databases (29) for *E. coli* strains. The ResFinder database identified a higher number of AMR genes meeting the >90% threshold for coverage and identity, with the detailed heatmap presented in Fig. 10. For the detection of virulence factors, the Abricate tool was also employed to compare against the Virulence Factor Database (VFDB) (http://www.mgc.ac.cn/VFs/). Matches surpassing the >90% threshold for coverage and identity were considered significant. Amino acid sequences from the six UPEC strains, including both the morphotype-positive and negative UPEC strains, were analyzed using the BLAST software to compare them against the VFDB.

### Sequencing data availability

The sequencing data of MLST types and the WGS data reported in this paper have been deposited into the sequence read archive (SRA) database under the NCBI BioProject accession numbers PRJNA1165699 and PRJNA1145961, respectively.

### Statistical analysis

The CFU counts of the urine sample before and after urine vortexing treatment for both the MP group and MN group were analyzed statistically. Furthermore, the results of all virulence genes detected by PCR in this study, phylogenetic groups, and MLST of the two groups were obtained for analysis. Statistical significance was determined using the SPSS version 21.0 for Windows (SPSS Inc., Chicago, IL, USA) through the analysis of data with the non-parametric Mann-Whitney test, the Chi-square test, and the Fisher exact test. The threshold for statistical significance was set at a *P* value < 0.05.

## RESULTS

### Composition of pathogenic bacteria in UTIs

A total of 184 pathogenic bacteria were isolated from the urine of 535 patients with urinary tract infections, resulting in a urine culture positivity rate of 34.39%. Among these bacteria, seven different species were identified. *Escherichia coli* was the most prevalent, representing 77.17% (142 strains), followed by *Klebsiella pneumoniae* (9.24%, 17 strains), *Enterococcus faecalis* (4.89%, 9 strains), *Streptococcus agalactiae* (4.35%, 8 strains), and *Proteus mirabilis* (3.26%, 6 strains). Additionally, there was one case each of *Pseudomonas aeruginosa* and *Staphylococcus aureus*, both accounting for 0.54%.

### A high proportion of MP group revealed by bright microscope

**Fig 2:**
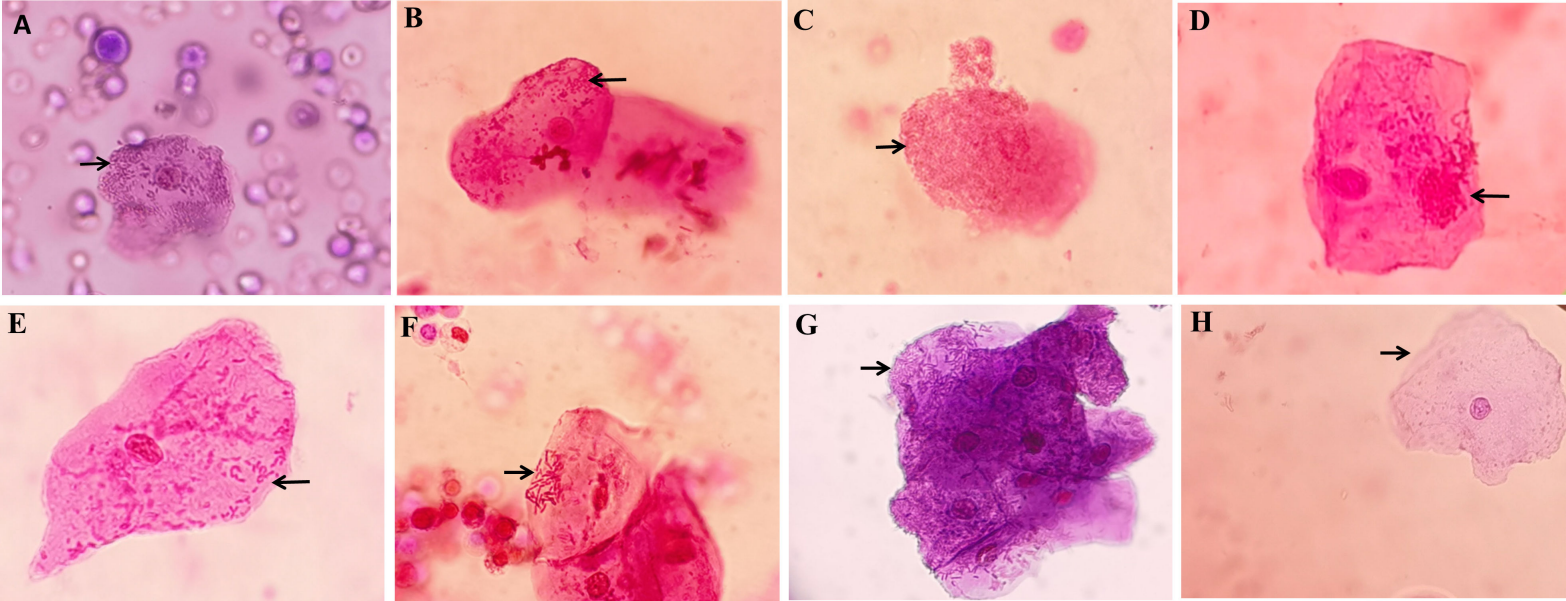
The morphotypes of UPEC observed in exfoliated urothelial cells by a light microscope (40×). The urine sediment was stained by Sternheimer-Malbin. Among the eight images, all except image (**H**) represent MP UPEC. (**A**, **B**, **E**, **F**, and **G**) The scattered UPEC adheres to urinary epithelial cells, being an intracellular UPEC. (**C**) Biofilm. (**D**) An intracellular bacterial community (IBC), a large number of bacteria that accumulate and form compact clusters of bacteria that can be observed in epithelial cells. (**H**) No UPEC was observed within the epithelial cells, which was classified as MN bacteria. UPEC, uropathogenic *Escherichia coli*; MP, morphotype positive; MN, morphotype negative.

**Fig 3:**
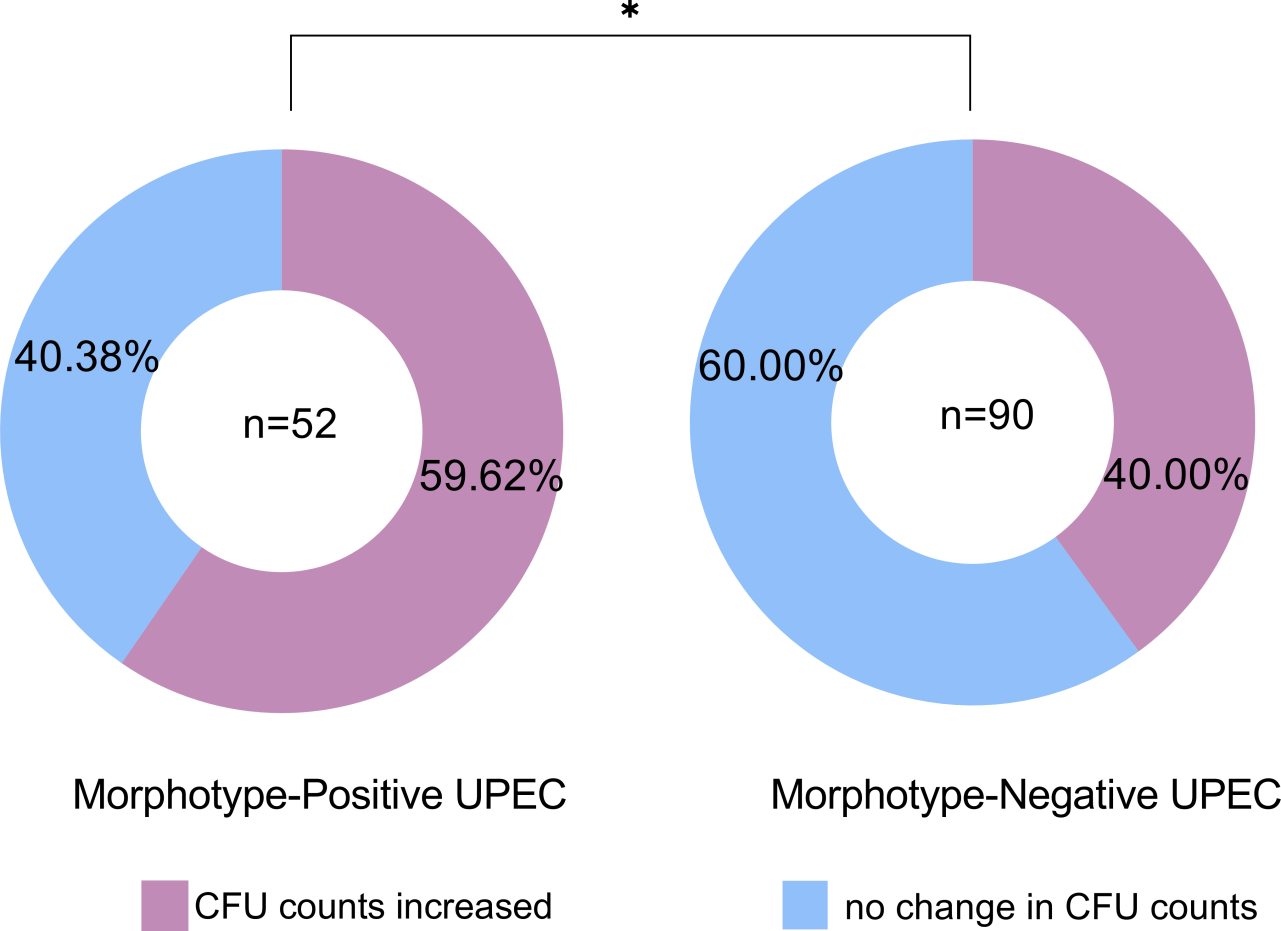
Comparison of the CFU counts before and after vortexing between the urine with morphotype-positive and negative UPEC. Urine samples with the morphotype-positive and negative UPEC were all subjected to urine culture after vortexing. CFU values increased in 59.62% of the MP UPEC and in 40.00% of the MN UPEC. A significant difference was observed between the two groups, indicating that the MP UPEC affected the CFU value greatly (χ^2^ = 5.089, *P* = 0.024). Statistical analysis was performed using the Chi-square test, and analyses were conducted using the SPSS version 21.0 for Windows (SPSS Inc., Chicago, IL, USA). *P* < 0.05 marked as *. UPEC, uropathogenic *Escherichia coli*; MP, morphotype positive; MN, morphotype negative.

Out of the 142 isolates of *Escherichia coli*, 52 belonged to the MP group (36.62%), while the remaining 90 isolates belonged to the MN group. The morphotypes of adherence and IBC are illustrated in Fig. 2. Following vortex application, the urine samples from the MP group were subjected to urine culture, revealing increased CFU values in 31 cases (59.62%). In contrast, CFU values increased in 36 cases (40.00%) in the MN group. A statistically significant difference was observed between the MP and MN groups after the vortex application (χ^2^ = 5.089, *P* = 0.024) (Fig. 3).

### A high percentage of the phylogenetic group B2 in both the MP and MN groups

**Fig 4:**
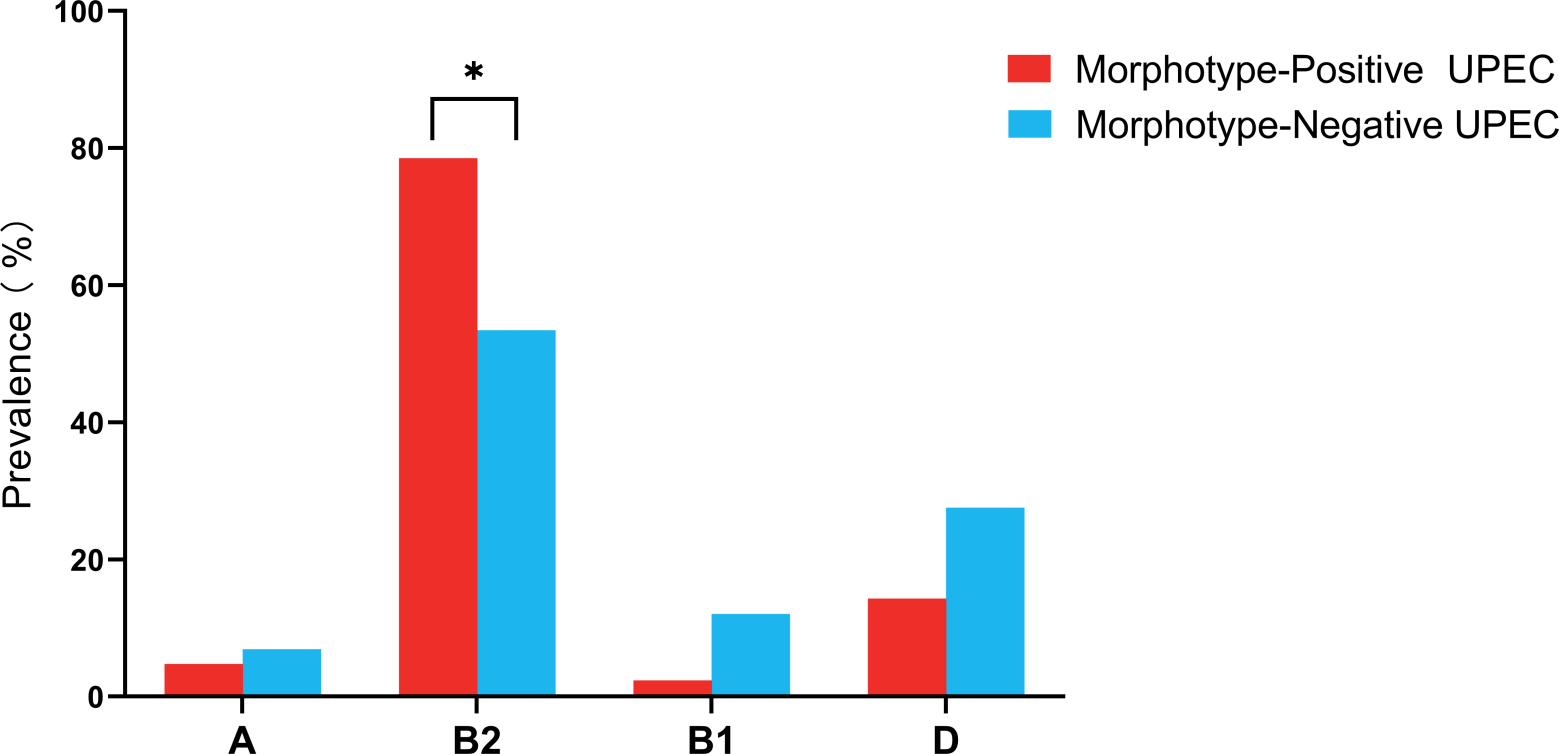
Comparison of the phylogenetic group between the morphotype-positive and negative UPEC. The figure displays the distribution of different phylogenetic groups (A, B1, B2, and D) among MP (*n* = 42) and MN (*n* = 58) UPEC isolates. Group A: the prevalence in MP UPEC was 4.76% compared to 6.90% in MN UPEC, with a *P* value of 0.750. Group B1: the prevalence in MP UPEC was 2.38% compared to 12.07% in MN UPEC, with a *P* value of 0.134. Group B2: the prevalence in MP UPEC was 78.57%, significantly higher than the 53.45% observed in MN UPEC, with a *P* value of 0.010. Group D: the prevalence in MP UPEC was 14.29% compared to 27.59% in MN UPEC, with a *P* value of 0.113. Statistical analysis was performed using the Chi-square test, and analyses were conducted using the SPSS version 21.0 for Windows (SPSS Inc., Chicago, IL, USA). B2, phylogenetic group B2; B1, phylogenetic group B1; A, phylogenetic group A; D, phylogenetic group D; MP, morphotype positive; MN, morphotype negative.

The phylogenetic group B2 was the most prevalent within both the MP and MN groups, accounting for 78.57% of the MP group and 53.45% of the MN group, respectively (Fig. 4; Table S3). This result was statistically significant (*P* = 0.010), with more virulence genes detected in the MP group. This finding aligns with the established understanding that the phylogenetic group B2 of UPEC is associated with a higher number of virulence genes. These virulence genes are particularly relevant due to their roles in enhancing bacterial adhesion, a critical factor in UPEC pathogenicity (Fig. 7).

### Multilocus sequence typing analysis

**Fig 5:**
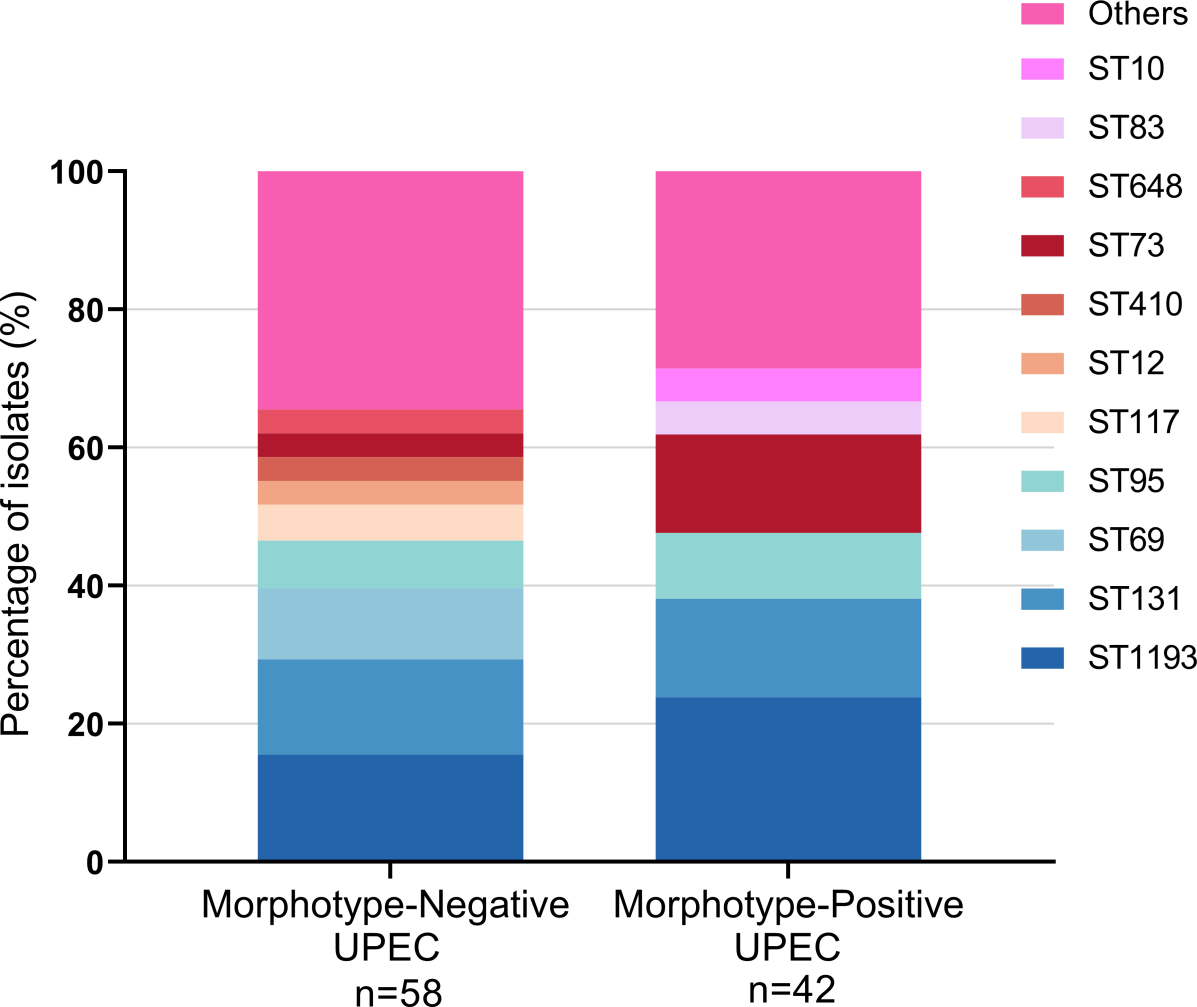
Comparison of the prevalence of MLST types between the morphotype-positive and negative UPEC. The prevalence of nine prominent STs was assessed in two groups, with those occurring only once, grouped as "others." The most prevalent STs in the MP group were ST1193 (23.81%) and ST131 (14.29%), followed by ST73 (14.29%), ST95 (9.52%), ST83 (4.76%), and ST10 (4.76%). Similarly, in the MN group, the top ST was ST1193 (15.52%), followed by ST131 (13.79%), ST69 (10.34%), ST95 (6.90%), ST117 (5.17%), and ST12 (3.45%). ST1193 and ST131 were common in both groups, and there was no significant difference in the phylogenetic group between the two groups. Statistical analysis was performed using the Chi-square test, and analyses were conducted using the SPSS version 21.0 for Windows (SPSS Inc., Chicago, IL, USA). MLST, multilocus sequence typing; UPEC, uropathogenic *Escherichia coli*; MP, morphotype positive; MN, morphotype negative.

The sequence types ST1193 and ST131 were prevalent in both groups. ST1193 was found in 23.81% of the MP group and 15.52% of the MN group, with no statistically significant difference (*P* = 0.297). Similarly, ST131 constituted 14.29% of the MP group and 13.79% of the MN group, also showing no significant difference (*P* = 0.944). The lack of significant differences across these nine sequence types indicates that no clear discrimination can be made between the morphotypes (Fig. 5; Table S5).

### Enhanced virulence gene profiles in the MP group

**Fig 6:**
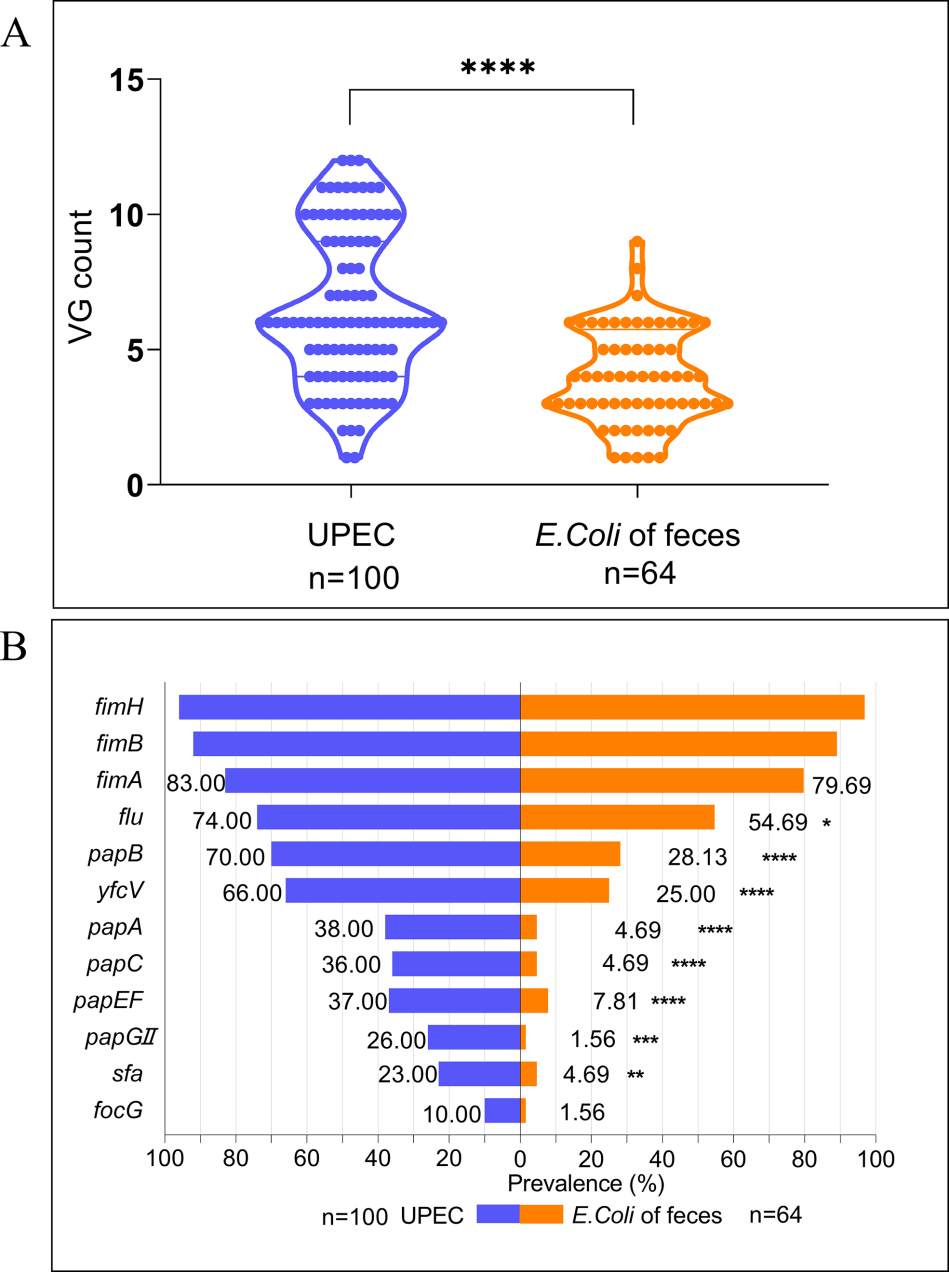
(A) Total number of VG harboring per strain for *E. coli* isolates from urine and feces. The statistical analysis was performed using the non-parametric Mann-Whitney test, and the analyses were conducted using the SPSS version 21.0 for Windows (SPSS Inc., Chicago, IL, USA). (B) Prevalence of 12 adhesion-related VGs for the *E. coli* isolates from urine and feces. The statistical analysis was performed using the Chi-square test, and the analyses were conducted using the SPSS version 21.0 for Windows (SPSS Inc., Chicago, IL, USA). *E. coli* from urine samples showed a higher prevalence of adhesion-related VGs compared to fecal samples, with median VG counts of 6 (IQR 4–10) and 4 (IQR 3–6), respectively. Genes such as *papGII, papC, papA, sfa, papEF, yfcV, papB,* and *flu* were significantly more prevalent in urine isolates. This indicates that *E. coli* from feces with more adhesion VGs is more likely to colonize the urinary tract and cause infections. Significance levels: *P < 0.05, **P < 0.01, ***P < 0.001, ****P < 0.0001. VG, virulence gene.

**Fig 7:**
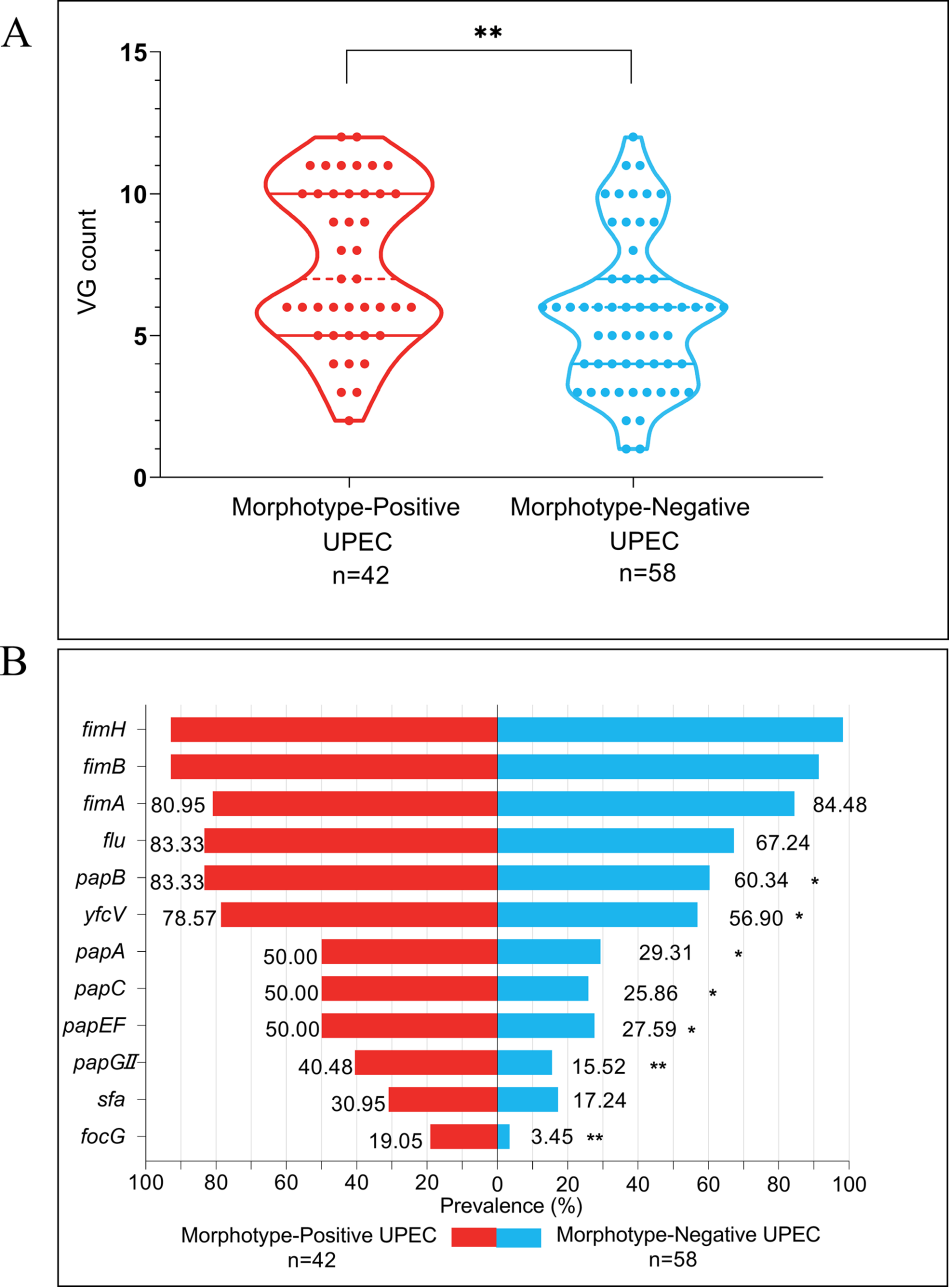
(A) Total number of VG harboring per strain for *E. coli* isolates among the MP and MN UPEC. The statistical analysis was performed using the non-parametric Mann-Whitney test, and the analyses were conducted using the SPSS version 21.0 for Windows (SPSS Inc., Chicago, IL, USA). (B) Prevalence of 12 adhesion-related virulence genes for the *E. coli* isolates among the MP and MN UPEC. The statistical analysis was performed using the Chi-square test, and the analyses were conducted using the SPSS version 21.0 for Windows (SPSS Inc., Chicago, IL, USA). MP UPEC showed a higher prevalence of adhesion-related VGs compared to MN UPEC, with median VG counts of 7 (IQR 5–10) and 6 (IQR 4–7), respectively. Notably, genes such as *papGII*, *papC, papA, papEF, yfcV, papB*, and *focG* were significantly more prevalent in the MP UPEC. This suggests that adhesion genes associated with P fimbriae may play a more important role in the invasion of epithelial cells forming the MP UPEC. Significance levels: *P < 0.05, **P < 0.01, ***P < 0.001, ****P < 0.0001. VG, virulence gene; MP, morphotype positive; MN, morphotype negative.

We first compared the adhesion-related virulence genes between *E. coli* isolated from the urine and feces. A higher prevalence of adhesion-related virulence genes in *E. coli* isolated from the urine was revealed. *E. coli* isolated from the urine had a median VG count of 6 (interquartile range [IQR] 4–10), whereas *E. coli* isolated from the feces exhibited a lower median VG count of 4 (IQR 3–6), as shown in Figure 6 and Table S5. Furthermore, Figure 7 depicts the distribution of VGs per strain within the MP and MN groups. A statistically significant difference in VG counts was noted between the EC-MP (7, IQR 5–10) and EC-MN (6, IQR 4–7) groups (*P* = 0.0029). The MP group also displayed a significantly higher prevalence of specific virulence genes, and these results (Fig. 7; Table S6) underscore the enhanced pathogenic potential of UPEC belonging to the MP group.

### Susceptibility profiles of the MP and MN groups to β-Lactam antibiotics and other drug classes

The susceptibility profiles of strains from the MP and MN groups to β-lactam antibiotics exhibited significant variations. Specifically, resistance to ampicillin was observed at 69.05% in the MP strains and 75.86% in the MN strains. For cefuroxime, resistance was 28.57% in the MP strains compared to 43.10% in the MN strains. The same resistance percentages were found for ceftriaxone in the MP strains (28.57%) and MN strains (43.10%). Notable differences were also identified in resistance to other antibiotics: ceftazidime (4.76% MP vs 15.52% MN), cefoxitin (2.38% MP vs 12.07% MN), and cefepime (9.52% MP vs 24.14% MN).

In terms of β-lactamase inhibitor combinations, resistance to amoxicillin/clavulanate was slightly higher in the MN strains (10.34%) compared to the MP strains (2.38%). Similarly, resistance to cefoperazone/sulbactam was 2.38% in the MP strains and 1.72% in the MN strains, while piperacillin/tazobactam resistance was 2.38% in the MP strains versus 3.45% in the MN strains.

**Fig 8:**
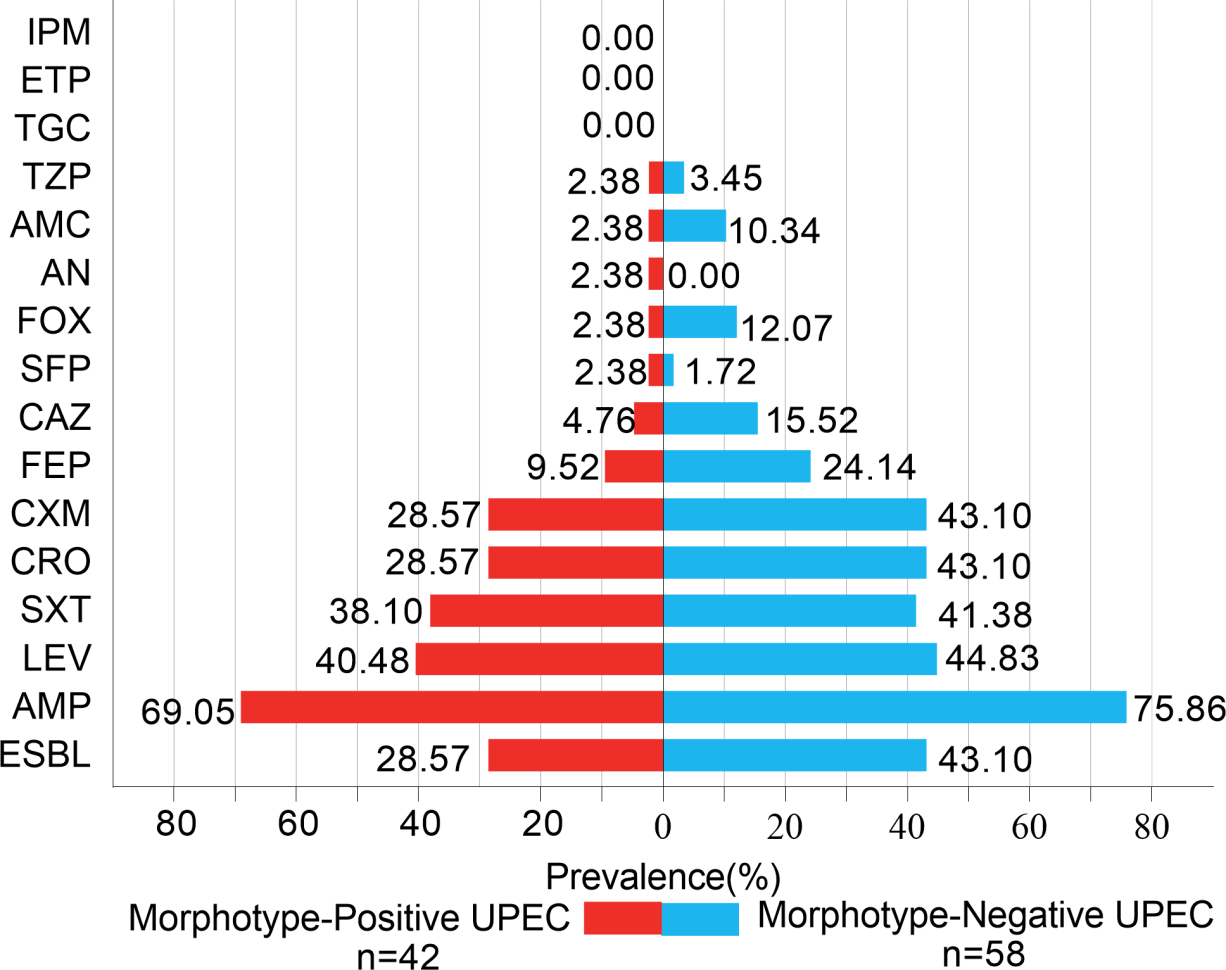
The result of antibiotic resistance among the MP and MN UPEC. Forty-two MP UPEC and 58 MN UPEC strains were tested for antibiotic resistance to 15 antibiotics. All strains were highly susceptible to a glycylcycline class of semisynthetic antimicrobial agent (Tigecycline) and carbapenems (Imipenem and Ertapenem), which are beta-lactams. MP UPEC strains show similar resistance as MN UPEC strains, and no significance was analyzed between the two groups, indicating that MP UPEC strains may not contribute to increased resistance rates and pose a threat to antibiotics. Statistical analysis was performed using the Chi-square test and the Fisher exact test, and all analyses were conducted using the SPSS version 21.0 for Windows (SPSS Inc., Chicago, IL, USA). UPEC, uropathogenic *Escherichia coli*; IPM, imipenem; ETP, ertapenem; TGC, tigecycline; TZP, piperacillin/tazobactam; AMC, amoxicillin/clavulanic acid; AN, amikacin; FOX, cefoxitin; SFP, cefoperazone/sulbactam; CAZ, ceftazidime; FEP, cefepime; CXM, cefuroxime; CRO, ceftriaxone; SXT, trimethoprim/sulfamethoxazole; LEV, levofloxacin; AMP, ampicillin; ESBL, extended-spectrum β-lactamases; MP, morphotype positive; MN, morphotype negative.

Both the MP and MN strains showed a high sensitivity to sulfa drugs, with compound sulfamethoxazole resistance at 2.38% in the MP strains and no resistance in the MN strains. Additionally, both groups were highly susceptible to carbapenems (ertapenem and imipenem) and the glycylcycline antibiotic tigecycline. Resistance to the aminoglycoside amikacin was also noted, with 38.10% resistance in the MP strains and 41.38% in the MN strains (Fig. 8; Table S7).

### Comparative analysis of virulence genes and antibiotic resistance in the MP and MN UPEC groups by whole genome sequencing

A comparative analysis of virulence genes between the MP strains (GN115, GN554, GN100) and MN strains (GN439, GN471, GN531) revealed that the MP strains possessed a higher number of virulence genes, particularly in categories such as adhesins, immune modulators, toxins, nutritional and metabolic factors, and effector delivery systems. The MP strains exhibited strong adherence capabilities due to an abundance of adhesion-related genes, and they also contained significant numbers of genes associated with nutrition, metabolism, invasion, and effector delivery systems. Notably, all three MP strains shared toxin-related virulence genes, and strain GN115 had a unique immune modulation-related gene. However, none of the MP strains contained virulence genes linked to the Lee pathogenicity island.

**Fig 9:**
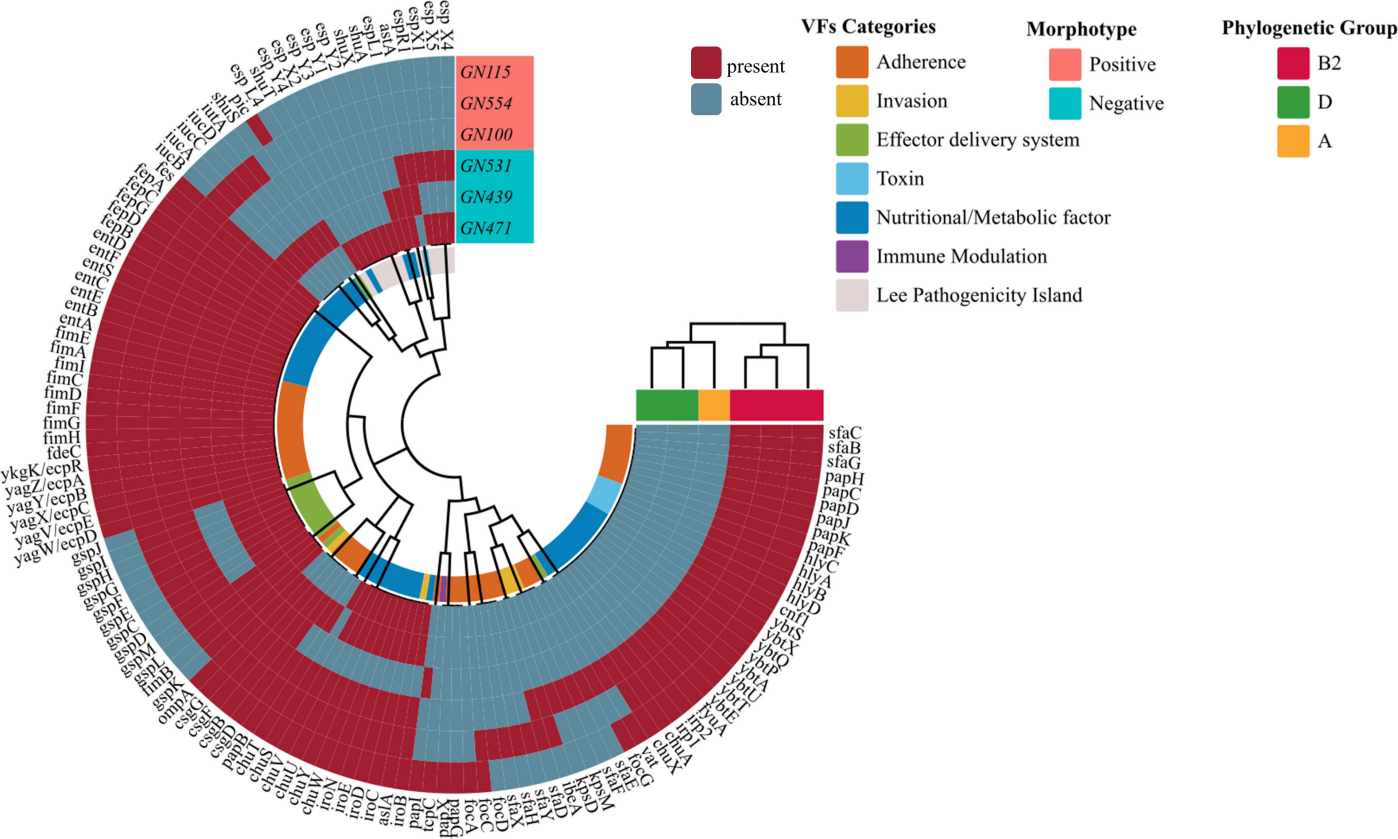
Virulence genes detected by whole genome sequencing for selected strains with MP and MN UPEC. The different phylogenetic groups, VF categories, and morphotypes are color coded and illustrated at the tips. The occurrence of virulence genes is also coded by two different colors. The outer three rings in this circular diagram represent the three MP UPEC strains, which carry significantly more virulence genes compared to the three MN UPEC strains represented by the inner three rings. This indicates that the positive strains have stronger virulence than the negative strains. MP, morphotype positive; MN, morphotype negative; UPEC, uropathogenic *Escherichia coli.*

In contrast, the MN strains (GN439, GN471, GN531) showed lower counts of adhesion-related genes, invasion-related genes, and genes associated with effector delivery systems. The number of toxin-related virulence genes in these strains was 1, 0, and 1, respectively. The counts for genes related to nutrition and metabolism were 31, 26, and 13, respectively. Additionally, strain GN439 carried the espL1 gene from the *Esp* series, which is linked to the Lee virulence gene island. Strain GN471 contained 11 genes from this series (*espL1*, *espX1*, *espR1*, *espX5*, *espX4*, *espL4*, *espY4*, *espX2*, *espY3*, *espY1*, *espY2*), while strain GN531 harbored five genes from the same series (*espL1*, *espX1*, *espR1*, *espX5*, *espX4*) (Fig. 9).

**Fig 10:**
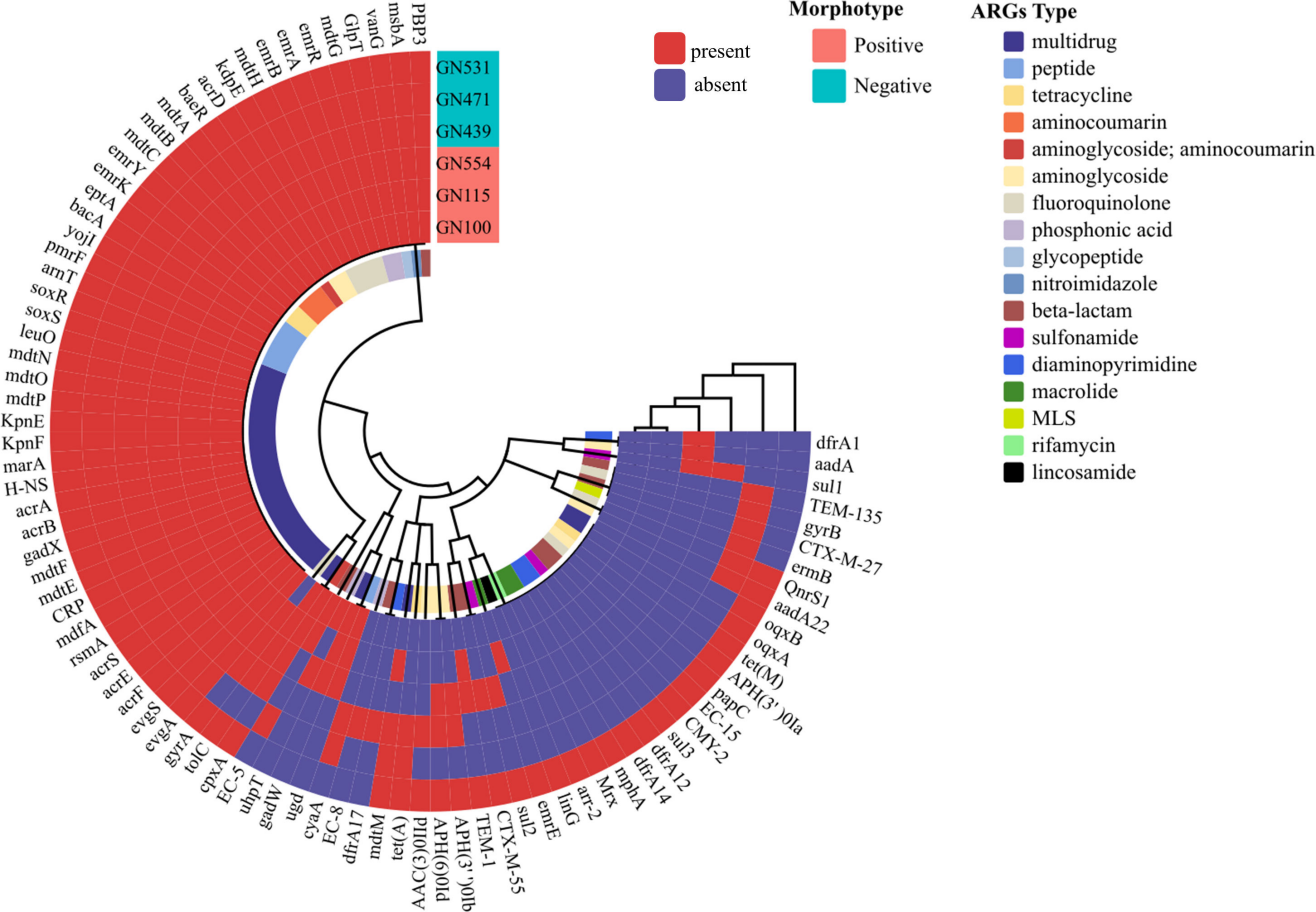
Antibiotic resistance genes detected by whole genome sequencing for selected strains with MP and MN UPEC. The various categories of resistance genes, morphotypes, and phylogenetic groups are color coded and depicted at the tips. The occurrence of antibiotic resistance genes is also coded by two different colors. The outer three rings in this circular diagram represent the three MP UPEC strains, and the inner three rings represent the MN UPEC strains. Apart from the GN531 strain, which carries the highest number of resistance genes, the MP and MN UPEC strains show similar profiles in terms of resistance genes carried. MP, morphotype positive; MN, morphotype negative; UPEC, uropathogenic *Escherichia coli.*

Furthermore, a total of 17 antibiotic resistance phenotypes and 87 drug resistance genes were detected in the MP and MN UPEC strains. Multidrug resistance was the most prevalent drug resistance phenotype in both groups. There was no significant difference in the number of resistance genes between the two groups (Fig. 10).

## DISCUSSION

Previous studies have emphasized the importance of the bacterial morphotype of UPEC in recurrent urinary tract infections (30–33). Yet, there is a lack of literature discussing the influence of the bacterial morphotype of UPEC on the result of urine culture, particularly in observing the variations in urine culture results pre- and post-vortexing. Additionally, the molecular epidemiological distinctions between MP and MN UPEC remain unclear.

This study found that the proportion of UPEC with bacterial morphotype accounted for a large number, although slightly lower than the proportion observed in a study conducted outside of our region, as described in reference (34). The latter used both optical microscopy and confocal microscopy, but in this study, we performed only optical microscopy, which may be the most important reason for the difference in this proportion. Moreover, the CFU count in the urine samples carrying MP UPEC was found to be lower compared to those with MN UPEC. These results are in agreement with those obtained in earlier studies for the presence of IBC, which can lead to low or even negative CFU results in urine culture (34). A possible explanation for this might be the UPEC that adhered to epithelial cells, being intracellular bacteria causing a decrease in the concentration of UPEC in urine. The most important result was that after vortexing, the urine samples carrying the bacterial morphotype of UPEC had higher CFU values than those that did not carry it, and the difference was statistically significant. This result ties well with an earlier observation, which showed the phenomenon of increased CFU was also found in Mexico in 2021 (14). Unfortunately, the study from Mexico did not group urine samples according to bacterial morphotype, so complete comparisons cannot be made here. This interesting finding could be due to a certain number of bacteria adhered to the urothelial cells being released into the urine by way of vortexing. A recent study determined that a single epithelial cell containing an early IBC typically harbors 10^3^ viable bacteria (31). It is worth noting that the CFU count in some urine samples without bacterial morphotypes also increased after vortexing. This finding may be somewhat limited by the sole use of an optical microscope in our study, which may have reduced sensitivity in detecting adhesion, IBC, and filamentous structures, potentially resulting in false negatives for bacterial morphotypes.

Our study revealed a significant disparity in the prevalence of phylogenetic group B2 between MP UPEC and MN UPEC, suggesting that MP UPEC may harbor a larger repertoire of virulence genes (35, 36), particularly those related to adhesion. Through both PCR and whole genome sequencing, we confirmed that MP UPEC possessed a higher prevalence of adhesion-related virulence genes compared to MN UPEC, especially those associated with type 1 fimbriae. This finding supports previous studies indicating their crucial role of type 1 fimbriae in facilitating bacterial adhesion to urinary epithelial cells (9). Moreover, the high prevalence of *fimH* and *papGII* genes detected in the MP group with strong invasion supports the observation that fimH adhesins work synergistically with papGII adhesins to enhance the establishment and maintenance of infection (37). As illustrated in Fig. 9, MP UPEC not only exhibited a greater variety of adhesion-related genes but also demonstrated an overall higher presence of virulence genes related to both adhesion and invasion compared to MN UPEC, further highlighting the pathogenic potential of MP UPEC. However, a study by Robino et al. found no significant differences in virulence genes between IBCs/IIB and non-IBC/IIB UPEC isolated from children (38). An explanation for this possibly is the different immunity between adults and children, resulting in the different virulence of pathogens (39). Additionally, our study observed a significant disparity in adhesion-related virulence genes between UPEC strains and *E. coli* from the feces of healthy volunteers. A higher average number of these genes per UPEC strain harboring, as well as a higher prevalence, were observed, which aligns with the findings by Rezatofighi et al. (40). This suggests that *E. coli* with more adhesion-associated virulence genes is more likely to colonize the urinary tract and cause UTIs, supporting the idea that rectal flora acts as a reservoir for these infections (41).

In this study, both groups of UPEC strains exhibited high resistance to β-lactam antibiotics, such as ampicillin, while maintaining high susceptibility to carbapenems, including ertapenem and imipenem, as well as tetracyclines like tigecycline and aminoglycosides such as amikacin. These findings are consistent with a 2021 study from Korea (42), which reported similar resistance patterns, although resistance levels were generally higher in the Korean study. This difference may be attributed to the fact that the majority of UPEC strains in the Korean study belonged to phylogenetic group B2, which is known for its enhanced antibiotic resistance (43, 44). Additionally, those strains were isolated from patients with chronic conditions such as cancer, hepatitis, and cirrhosis, who often receive long-term medication treatments, potentially contributing to the increased resistance (45, 46). WGS of some strains supported the susceptibility testing results to some extent. Interestingly, despite the presence of multiple resistance genes, such as *evgS*, *tolC*, and others in five of the six sequenced isolates, these strains were mostly susceptible to the 13 antibiotics tested. This discrepancy may be due to several factors: the resistance genes may be underexpressed or not expressed under the current laboratory conditions, and without selective pressure from antibiotics, these genes may remain inactive. These factors highlight the complexity of correlating genetic resistance determinants with phenotypic resistance (47). Notably, a previous study (48) suggested that IBCs might protect bacteria from antibiotics and the host immune system. However, our study found no significant differences in antibiotic resistance between the two UPEC groups based on susceptibility testing. It is possible that the dispersed rod-shaped UPEC does not pose a significant threat of antibiotic resistance. This highlights the complexity of resistance mechanisms and suggests that further research is needed to understand the role of bacterial morphology and host factors in antibiotic resistance.

This study highlights the diagnostic challenges posed by UPEC strains exhibiting positive bacterial morphotypes. Our findings are consistent with prior studies suggesting that UPEC can adopt intracellular forms to evade detection in standard urine cultures (11, 12). These morphological changes, while transient, significantly impact diagnostic accuracy and may lead to false-negative urine culture results, creating a critical gap in diagnostic sensitivity. However, it is worth noting that among the three stages of morphological changes, while adhesion and IBCs were effectively observed in this study, filamentous UPEC was not clearly observed, which may limit the completeness of our analysis. This may be attributed to the filamentation response being contingent upon the mild acidity of human urine and specific components within the low molecular-mass fraction derived from a mildly dehydrated individual (49).

Our study contributes to the understanding of how bacterial morphotypes interfere with traditional diagnostic workflows. By demonstrating the substantial increase in CFU counts after vortexing, this study highlights the necessity of standardizing vortexing in clinical diagnostics to reduce false-negative results and prevent misdiagnosis or inappropriate treatment. While vortexing is widely recognized in laboratory settings globally, its diagnostic value remains underexplored. In China, the National Clinical Laboratory Procedures do not explicitly include this step, and its role in improving culture positivity is often overlooked in clinical practice. Standardizing this method in China could address these gaps and enhance diagnostic accuracy, as current guidelines may not fully reflect its importance.

For instance, patients with MP UPEC may have symptoms of UTIs but show negative urine culture results. This can lead to misdiagnosis of conditions like urolithiasis (50). Moreover, false-negative results may make doctors stop antibiotics too soon, increasing the risk of recurring UTIs and contributing to antimicrobial resistance.

Globally, the findings of this study have important implications for improving diagnostic practices. In regions where urine culture remains the gold standard for UTI diagnosis, adopting vortexing and recognizing the impact of bacterial morphotypes can enhance diagnostic sensitivity, reduce treatment delays, and improve patient outcomes. Furthermore, our study provides a framework for future research into the role of bacterial morphotypes in other diagnostic challenges, such as persistent or recurrent infections. By addressing these gaps, we aim to bridge the divide between laboratory findings and clinical applications, promoting evidence-based improvements in UTI management.

Although this study provided valuable insights into the impact of morphotype-positive UPEC on urine culture outcomes and revealed important molecular epidemiological differences, there are still some limitations that should be addressed in future research. In this study, 36.62% of MP UPEC was detected using brightfield microscopy, which is a lower detection rate compared to fluorescence or electron microscopy. This lower sensitivity of brightfield microscopy may increase the risk of missed detections. Furthermore, the increase in CFU counts of the MN group by 40.00% in the study was attributed to a certain number of grouping errors, also by equipment restriction mentioned above. Nevertheless, the 36.62% detection rate indeed confirmed the importance of brightfield microscopy in bacterial morphotype detection. Meanwhile, the significant difference between the MP and MN groups post-vortex application indicated the effectiveness of urine vortexing in increasing CFU counts. Therefore, for laboratories lacking advanced equipment, brightfield microscopy and urine vortexing may represent a simple and cost-effective method. Additionally, the urine samples in this study were obtained from the same research center, suggesting that future research should consider a multicenter approach for a comprehensive molecular epidemiological study.

In the present study, we have reported the prevalence of UPEC with bacterial morphotype and established a direct relationship between urine culture positivity rate and bacterial morphotype (14). For the first time, we demonstrated the visual changes in CFU values of urine samples carrying the morphotype UPEC before and after vortexing was presented. In this regard, a promising future direction for UTI treatment could be the development of vaccines targeting UPEC, particularly fimbriae involved in bacterial adhesion. By inhibiting UPEC’s ability to adhere to and invade epithelial cells, these vaccines may reduce the formation of intracellular bacteria, thereby decreasing the number of morphotype-positive UPEC. When combined with appropriate antibiotic therapy, this strategy has the potential to more effectively eradicate UPEC and reduce the recurrence of UTI. In addition, the molecular epidemiological distinctions between morphotype-positive and negative UPEC isolated from the urine of adults were revealed by our data. To further clarify these findings, detailed strain-by-strain data—including antibiotic susceptibility profiles, virulence genes, and phylogenetic groups for all 100 *E. coli* isolates—are available in the Fig. S1, ensuring full transparency of the data. As shown in Fig. S1, the two most prevalent sequence types of morphotype-positive and negative *E. coli* exhibit homology. Notably, most morphotype-positive *E. coli* belongs to phylogenetic group B2 and harbors more virulence genes than morphotype-negative *E. coli*, indicating stronger pathogenicity, although there was no significant difference in antibiotic sensitivity between the two groups. These findings highlight the importance of considering bacterial morphotype in the laboratory diagnosis of UTI, suggesting that greater emphasis should be placed on bacterial morphotype in clinical practice.

### Conclusion

When a number of intracellular bacteria or IBC or even filamentous bacteria adhering to epithelial cells are observed, it is necessary to perform a routine urine bacterial culture after releasing these bacteria through vortexing, to improve the positivity rate and reduce the risk of missed diagnosis. However, we recognize that due to the limitations of light microscopy, some UPEC adhering to the surface of epithelial cells may have been inadvertently grouped with intracellular bacteria as MP UPEC. Despite this limitation, light microscopy remains a widely accessible tool for the initial detection of bacterial morphotypes in most clinical laboratories, making our findings practically relevant. Moreover, bacterial morphotype can be combined with urine routine detection as an indicator to evaluate the treatment effect of urinary tract infection. Additionally, UPEC strains with bacterial morphotype carry more virulence genes and have stronger adhesion ability, but there is no threat to the selection of antibiotics for treatment.

## SUPPLEMENTAL MATERIAL

Supplemental tables and figure spectrum.00980-24-s0001.docx Tables S1 to S7; Figure S1.

ASM does not own the copyrights to Supplemental Material that may be linked to, or accessed through, an article. The authors have granted ASM a non-exclusive, world-wide license to publish the Supplemental Material files. Please contact the corresponding author directly for reuse.
