# Supplementary material for: Influence of bacterial morphotype on urine culture and molecular epidemiological differences in Escherichia coli harboring bacterial morphotype-induced urinary tract infections
Source: Microbiol Spectr. 2025 Mar 5;13(4):e00980-24. doi: 10.1128/spectrum.00980-24 (PMC11960099; doi:10.1128/spectrum.00980-24)
Supplement: Supplemental tables and figure — Tables S1 to S7; Figure S1. [file spectrum.00980-24-s0001.docx]

**Supplementary Material**

**Table S1** Primer sequences of genotyping of UPEC

| Analysis Marker Direction Sequence (5’一3’) SiZze Product (bp) | | | | |
| --- | --- | --- | --- | --- |
| MLST**^a,b^** | *adk* | Forward  Reverse | ATTCTGCTTGGCGCTCCGGG  CCGTCAACTTTCGCGTATTT | 583 |
|  | *fumC* | Forward  Reverse | TCACAGGTCGCCAGCGCTTO  GTACGCAGCGAAAAAGATTC | 806 |
|  | *gyrB* | Forward  Reverse | TCGGCGACACGGATGACGGC  ATCAGGCCTTCACGCGCATC | 911 |
|  | *icd* | Forward  Reverse | ATGGAAAGTAAAGTAGTTGTTCCGGCACA  GGACGCAGCAGGATCTGTT | 878 |
|  | *mdh* | Forward  Reverse | ATGAAAGTCGCAGTCCTCGGCGCTGCTGGCGG  TTAACGAACTCCTGCCCCAGAGCGATATCTTTCTT | 932 |
|  | *purA* | Forward  Reverse | CGCGCTGATGAAAGAGATGA  CATACGGTAAGCCACGCAGA | 816 |
|  | *recA* | Forward  Reverse | CGCATTCGCTTTACCCTGACC  TCGTCGAAATCTACGGACCGGA | 780 |
| phylogenetic grouping**^c^** | *gadA* | Forward  Reverse | GATGAAATGGCGTTGGCGCAAG  GGCGGAAGTCCCAGACGATATCC | 373 |
|  | *chuA* | Forward  Reverse | ATGATCATCGCGGCGTGCTG  AAACGCGCTCGCGCCTAAT | 281 |
|  | *yiaA* | Forward  Reverse | TGTTCGCGATCTTGAAAGCAAACGT  ACCTGTGACAAACCGCCCTCA | 216 |
|  | *TSPE4.C2* | Forward  Reverse | GCGGGTGAGACAGAAACGCG  TTGTCGTGAGTTGCGAACCCG | 152 |

**^a^** MLST, multilocus sequence typing

**^b^** the primers taken from the refference: Tartof SY, Solberg OD, Manges AR, Riley LW. Analysis of a uropathogenic Escherichia coli clonal group by multilocus sequence typing. J Clin Microbiol 2005;43:5860-4

**^c^** the primers taken from the refference: Yun KW, Kim DS, Kim W, Lim IS. Molecular typing of uropathogenic Escherichia coli isolated from Korean children with urinary tract infection. Korean J Pediatr. 2015 Jan;58(1):20-7. doi: 10.3345/kjp.2015.58.1.20. Epub 2015 Jan 31. PMID: 25729395; PMCID: PMC4342777.

**Table S2.** Primers sequence of twelve adhesion-related virulence genes

| Gene^d^ Sequence (5’-3’) Size Product (bp) | | | | |
| --- | --- | --- | --- | --- |
| *yfcV* | F: ACATGGAGACCACGTTCACC  R: GTAATCTGGAATGTGGTCAGG | 292 |  |  |
| *sfa* | F: CTCCGGAGAACTGGGTGCATCTTAC  R: CGGAGGAGTAATTACAAACCTGGCA | 410 |  |  |
| *fimH* | F: TGCAGAACGGATAAGCCGTGG  R: GCAGTCACCTGCCCTCCGGTA | 508 |  |  |
| *fimA* | F: AGTTAGGACAGGTTCGTACCGCAT  R: AAATAACGCGCCTGGAACGGAATG | 315 |  | |
| *fimB* | F: CGAATCACTCCTTAAAGCAG  R: GGCGTAACATGTGCGGATGAA | 379 |  |  |
| *papB* | F: GGGGAACCATGGCGCATCATGAAGTC  R: CTCCGGAATTCCATAATTTAGTCAAATG | 310 |  |  |
| *papGII* | F: GGGATGAGCGGGCCTTTGAT  R: CGGGCCCCCAAGTAACTCG | 190 |  |  |
| *papC* | F: CGGTTGGATTGTCAGCCTC  R: GCCATCAACCGGTACACCTC | 460 |  |  |
| *papA* | F: ATGGCAGTGGTGTCTTTTGGTG  R: CGTCCCACCATACGTGCTCTTC | 720 |  |  |
| *papEF* | F: GCAACAGCAACGCTGGTTGCATCAT  R: AGAGAGAGCCACTCTTATACGGACA | 336 |  | |
| *flu* | F: GGGTAAAGCTGATAATGTCG  R: GTTGCTGACAGTGAGTGTGC | 507 |  |  |
| *focG* | F: CAGCACAGGCAGTGGATACGA  R: GAATGTCGCCTGCCCATTGCT | 405 |  |  |

^d^ the primers taken from the refference:

1. Spurbeck RR, Dinh PC, Jr., Walk ST, et al. Escherichia coli isolates that carry vat, fyuA, chuA, and yfcV efficiently colonize the urinary tract. Infection and immunity. 2012;80(12):4115-4122.
2. Le Bouguenec C, Archambaud M, Labigne A. Rapid and specific detection of the pap, afa, and sfa adhesin-encoding operons in uropathogenic Escherichia coli strains by polymerase chain reaction. J Clin Microbiol. 1992;30(5):1189-1193.
3. Johnson JR, Stell AL. Extended virulence genotypes of Escherichia coli strains from patients with urosepsis in relation to phylogeny and host compromise. The Journal of infectious diseases. 2000;181(1):261-272.
4. Crépin S, Houle S, Charbonneau M, Mourez M, Harel J, Dozois CM. Decreased expression of type 1 fimbriae by a pst mutant of uropathogenic Escherichia coli reduces urinary tract infection. Infection and immunity. 2012;80(8):2802-2815.
5. Lane MC, Mobley HL. Role of P-fimbrial-mediated adherence in pyelonephritis and persistence of uropathogenic Escherichia coli (UPEC) in the mammalian kidney. Kidney international. 2007;72(1):19-25.

**Table S3.** The prevalence of phylogenetic groups among the morphotype-positive and negative UPEC

| phylogenetic group | morphotype-positive UPEC  n=42 (%) | morphotype-negative UPEC  n=58(%) | *P* value |
| --- | --- | --- | --- |
| A | 4.76 | 6.90 | 0.750 |
| B1 | 2.38 | 12.07 | 0.134 |
| B2 | 78.57 | 53.45 | 0.010 |
| D | 14.29 | 27.59 | 0.113 |

**Table S4.** The prevalence of sequence types among the morphotype-positive and negative UPEC

| ST | morphotype-positive UPEC  n=42 (%) | | morphotype-negative UPEC  n=58 (%) | | *P* value | |
| --- | --- | --- | --- | --- | --- | --- |
| ST1193 | 23.81 | 15.52  13.79  3.45  6.90  0.00  0.00 | | 0.297 | |  |
| ST131 | 14.29 |  |  | 0.944 | |  |
| ST 73 | 14.29 |  |  | 0.110 | |  |
| ST 95 | 9.52 |  |  | 0.917 | |  |
| ST 83 | 4.76 |  |  | - | |  |
| ST 10 | 4.76 |  |  | - | |  |
| ST 69 | 0.00 | 10.34 | | - | |  |
| ST117 | 5.17 | 0.00 | | - | |  |
| ST 12 | 5.17 | 0.00 | | - | |  |
| Others^a^ | 28.57 | 15.52 | | - | |  |

^a^ STs that occurred once time among morphotype-postive and morphotype-negative UPEC were categorized as “others”.

**Table S5.**  The prevalence of 12 adhesion-related virulence genes among UPEC and *E. coli* isolated from feces

| Gene | *E. coli* from urine  n=100(%) | *E. coli* from feces  n=64(%) | *P* value |
| --- | --- | --- | --- |
| *focG* | 10.00 | 1.56 | 0.074 |
| *papGⅡ* | 26.00 | 1.56 | 0.000038 |
| *papC* | 36.00 | 4.69 | 0.000004 |
| *papA* | 38.00 | 4.69 | 0.000002 |
| *sfa* | 23.00 | 4.69 | 0.002 |
| *papEF* | 37.00 | 7.81 | 0.000029 |
| *yfcV* | 66.00 | 25.00 | 0.000000 |
| *papB* | 70.00 | 28.13 | 0.000000 |
| *flu* | 74.00 | 54.69 | 0.011 |
| *fimA* | 83.00 | 79.69 | 0.592 |
| *fimB* | 92.00 | 89.06 | 0.524 |
| *fimH* | 96.00 | 96.88 | 0.771 |

**Table S6.**  The prevalence of 12 adhesion-related virulence genes among the morphotype-positive and negative UPEC

| Gene | morphotype-positive UPEC  n=42(%) | | morphotype-negative UPEC  n=58(%) | *P* value |
| --- | --- | --- | --- | --- |
| *focG* | 19.05 | 3.45 | | 0.010 |
| *papGⅡ* | 40.48 | 15.52 | | 0.005 |
| *papC* | 50.00 | 25.86 | | 0.013 |
| *papA* | 50.00 | 29.31 | | 0.035 |
| *sfa* | 30.95 | 17.24 | | 0.108 |
| *papEF* | 50.00 | 27.59 | | 0.022 |
| *yfcV* | 78.57 | 56.90 | | 0.024 |
| *papB* | 83.33 | 60.34 | | 0.013 |
| *flu* | 83.33 | 67.24 | | 0.070 |
| *fimA* | 80.95 | 84.48 | | 0.643 |
| *fimB* | 92.86 | 91.38 | | 0.788 |
| *fimH* | 92.86 | 98.28 | | 0.172 |

**Table S7.** Comparison of antibiotic resistance between morphotype-positive UPEC and morphotype-negative UPEC

| antibiotic | morphotype-positive UPEC  n=42  drug-resistance rate (%) | morphotype-negative UPEC  n=58  drug-resistance rate (%) | *P* value |
| --- | --- | --- | --- |
| AMP | 69.05 | 75.86 | 0.449 |
| LEV | 40.48 | 44.83 | 0.664 |
| SXT | 38.10 | 41.38 | 0.741 |
| CRO | 28.57 | 43.10 | 0.137 |
| CXM | 28.57 | 43.10 | 0.137 |
| FEP | 9.52 | 24.14 | 0.060 |
| CAZ | 4.76 | 15.52 | 0.090 |
| AN | 2.38 | 0.00 | 0.871 |
| AMC | 2.38 | 10.34 | 0.253 |
| TZP | 2.38 | 3.45 | 1.000 |
| SFP | 2.38 | 1.72 | 1.000 |
| FOX | 2.38 | 12.07 | 0.165 |
| ETP | 0.00 | 0.00 | - |
| IPM | 0.00 | 0.00 | - |
| TGC | 0.00 | 0.00 | - |


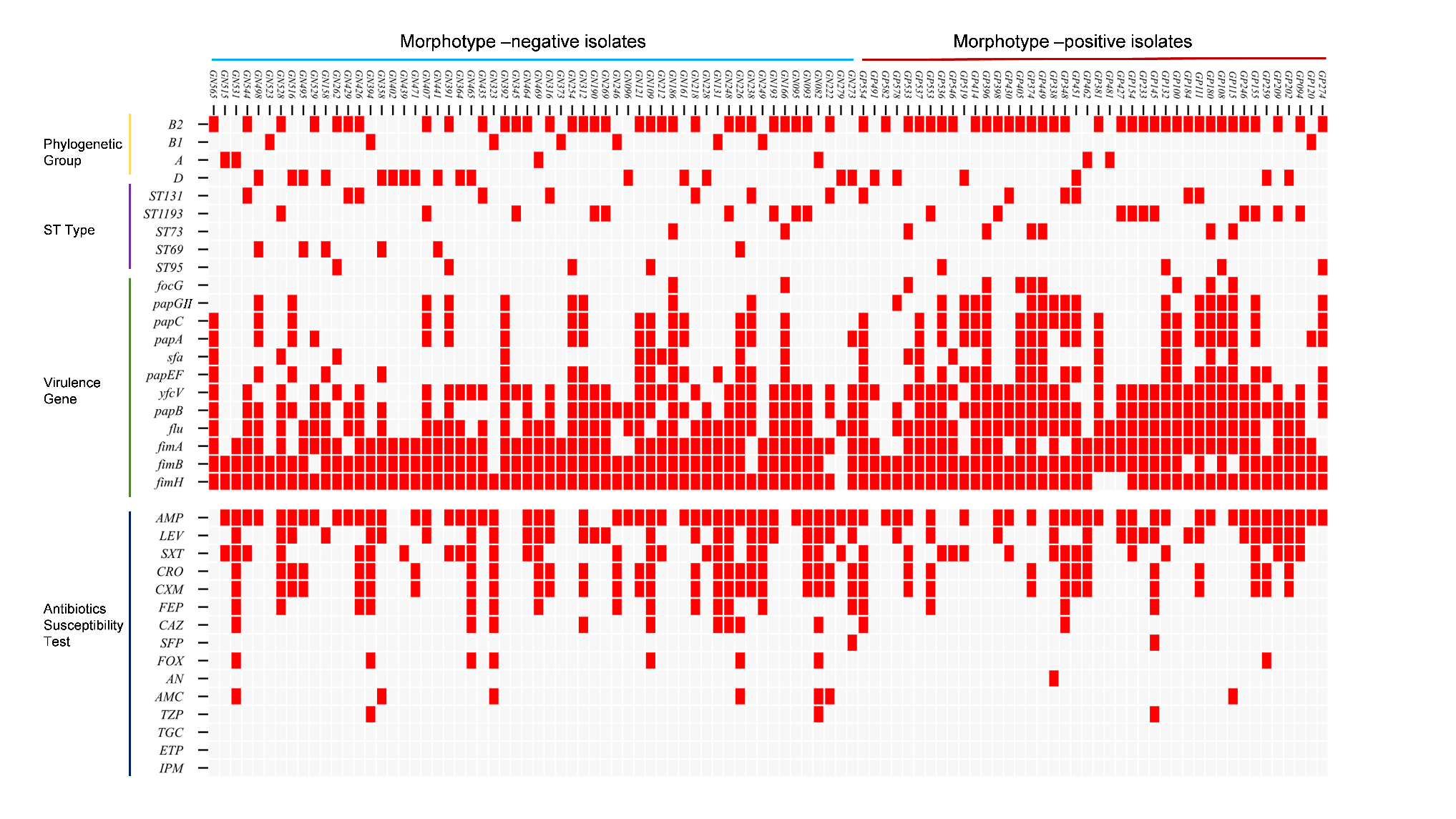


Figure S1. Virulence gene profiles, sequence types (ST), phylogenetic groups, and antibiotic susceptibility of 42 morphotype-positive and 58 morphotype-negative *Escherichia coli* isolates. The red blocks indicate the presence of virulence genes, specific ST types, or phylogenetic groups to which each isolate belongs. For the antibiotic susceptibility profiles, red blocks denote resistance, while white blocks indicate the absence of the corresponding gene or susceptibility to the antibiotic. The horizontal color bar represents the isolates, with morphotype-negative isolates on the left and morphotype-positive isolates on the right.
